# Epstein-Barr Nuclear Antigen 1 modulates replication of *oriP*-plasmids by impeding replication and transcription fork migration through the family of repeats

**DOI:** 10.1186/1743-422X-6-29

**Published:** 2009-03-05

**Authors:** Ashok Aiyar, Siddhesh Aras, Amber Washington, Gyanendra Singh, Ronald B Luftig

**Affiliations:** 1 Stanley S. Scott Cancer Center, LSU Health Sciences Center, 533 Bolivar Street, New Orleans, LA 70112, USA; 2 Department of Microbiology, LSU Health Sciences Center, 1901 Perdido Street, New Orleans, LA 70112, USA

## Abstract

**Background:**

Epstein-Barr virus is replicated once per cell-cycle, and partitioned equally in latently infected cells. Both these processes require a single viral *cis-*element, termed *oriP*, and a single viral protein, EBNA1. EBNA1 binds two clusters of binding sites in *oriP*, termed the dyad symmetry element (DS) and the family of repeats (FR), which function as a replication element and partitioning element respectively. Wild-type FR contains 20 binding sites for EBNA1.

**Results:**

We, and others, have determined previously that decreasing the number of EBNA1-binding sites in FR increases the efficiency with which *oriP*-plasmids are replicated. Here we demonstrate that the wild-type number of binding sites in FR impedes the migration of replication and transcription forks. Further, splitting FR into two widely separated sets of ten binding sites causes a ten-fold increase in the efficiency with which *oriP*-plasmids are established in cells expressing EBNA1. We have also determined that EBNA1 bound to FR impairs the migration of transcription forks in a manner dependent on the number of EBNA1-binding sites in FR.

**Conclusion:**

We conclude that EBNA1 bound to FR regulates the replication of *oriP*-plasmids by impeding the migration of replication forks. Upon binding FR, EBNA1 also blocks the migration of transcription forks. Thus, in addition to regulating *oriP *replication, EBNA1 bound to FR also decreases the probability of detrimental collisions between two opposing replication forks, or between a transcription fork and a replication fork.

## Background

Epstein-Barr virus (EBV) is replicated once per cell-cycle as an episome in proliferating latently infected cells [1,2]. Episomal replication requires a viral sequence *in cis*, termed *oriP*, and a single viral protein EBNA1 [3,4]. *OriP *contains two sets of binding sites for EBNA1, the region of dyad symmetry (DS), that contains four sites of low affinity for EBNA1, and the family of repeats (FR) that contains twenty high-affinity sites for EBNA1 [5,6]. DNA synthesis initiates at DS, in a manner dependent upon the association of the cellular origin recognition complex (ORC) proteins and minichromosome maintenance (MCM) proteins with DS [7-9]. Recent evidence indicates that EBNA1 recruits the ORC proteins to DS through an RNA-mediated interaction with ORC1 [10].

FR functions as a plasmid maintenance and partitioning element [11,12]. FR from the prototypic B95-8 strain of EBV contains 20 high-affinity sites for EBNA1, which binds each of these sites as a dimer [13,14]. EBNA1 bound to FR tethers viral episomes or *oriP *plasmids to cellular chromosomes [15-19]; an association that facilitates the plasmids to piggy-back into daughter cells at each metaphase [20,21]. In addition to its role in genome partitioning, two-dimensional gel analysis by Schildkraut and co-workers has indicated that the migration of replication forks through FR is attenuated, so that for the circular EBV genome or an *oriP*-plasmid, the bidirectional replication fork that initiates at DS is terminated at FR [22]. This ability of EBNA1 bound to FR to attenuate replication forks has been recapitulated in biochemical assays performed *in vitro*; such assays reveal that DNA binding domain of EBNA1 bound to FR impede the migration of replication forks from an SV40 origin on the same template [23].

Using assays for transcription activation and plasmid maintenance, we have examined the binding site requirements for EBNA1 in the EBV FR in detail [24]. Our analyses indicated that although the wild-type FR contains 20 binding sites, plasmids with 10 binding sites are maintained far more efficiently in colony formation assays than the former (*ibid*). A similar finding has been reported for deletion mutants constructed within the natural FR, in that a plasmid with nine binding sites replicated more efficiently than a plasmid with twenty binding sites [25]. Thus these results concur in that the wild-type number of EBNA1-binding sites in FR limits the replication of *oriP*-plasmids by acting *in cis*.

In this study, we have examined the mechanism by which the wild-type number of binding sites limits the replication of *oriP*-plasmids. Our results indicate that EBNA1 bound to FR limits replication by impeding the migration of replication forks from DS. In addition, we have determined that EBNA1 bound to FR severely impairs the migration of transcription forks through FR. We discuss both these findings in the context of the stable replication of EBV episomes.

## Methods

### Bacterial strains and plasmid purification

All plasmids were propagated in the *E. coli *strains DH5α, MC1061/P3, or STBL2 (Invitrogen, Carlsbad, CA). Plasmids used for transfection were purified on isopycnic CsCl gradients [26].

### Plasmids

Plasmids AGP73, and AGP74 have been described previously [24], and contain 10 and 20 EBNA1-binding sites in the FR respectively. These plasmids are constructed in the backbone of pPUR, and also contain EBV's DS and the EBV sequences between FR and DS. AGP81 contains 40 EBNA1-binding sites in FR and was constructed by dimerizing the FR in AGP74. AGP82 contains 80 EBNA1-binding sites in FR and was constructed by dimerizing the FR in AGP81. AGP83 has been described previously and is a control plasmid that only contains DS and completely lacks FR. AGP212, and AGP213 contain 20 EBNA1-binding sites split into two FRs each containing ten binding sites as described in the Results section. They were constructed as derivatives of AGP73. AGP212 was constructed by recovering an *Mfe*I-*Eco*RV fragment containing FR from AGP73 and inserting it into the *Eco*RI-*Bam*HI sites of that plasmid. AGP213 was constructed by inserting an *Eco*RV-*Acc*65I fragment from AGP73 into the *Acc65*I site of the same plasmid. Plasmid 2380 contains wild-type *oriP *cloned in pPUR, and was a gift from Bill Sugden. Plasmids AGP39, AGP40, and AGP41 were constructed as derivatives of pRSVL, by inserting 10, 20, or 40 EBNA1 binding sites between the end of the luciferase open reading frame and the SV40 polyadenylation signal in that plasmid. Plasmid 1606 has been described previously and expresses the large T antigen of SV40 under the control of the CMV immediate early promoter [27]. Plasmid 1160 has been described previously and expresses the DNA binding domain of EBNA1 under the control of the CMV immediate early promoter [28]. The empty expression vector, pcDNA3, was used as a control plasmid. Plasmid 2145 has been described previously and expresses EGFP under the control of the CMV immediate early promoter [17].

### Cell culture and transfections

The human cell line 293 [29], and its EBNA1-expressing derivative, 293/EBNA1, were used in this study. Both cell-types were grown in DMEM supplemented with 10% fetal bovine serum. G418 was added at a concentration of 200 mg/L to the media for 293/EBNA1 cells. Cells were grown at 37°C in a humidified 5% CO_2 _atmosphere. Plasmids were introduced into cells by the calcium phosphate method as described previously [17,18,24]. Transfections were normalized by the inclusion of a CMV-EGFP expression plasmid, 2145, in each transfection. Upon harvest, a fraction of the cells were profiled using a Becton-Dickinson FACSCalibur. Transfection efficiency was measured as the fraction of GFP-expressing, live cells quantified using CellQuest software from Becton-Dickinson (Franklin Lakes, NJ).

### Colony formation assays to assess plasmid maintenance and partitioning

Ten μg of AGP74 or an equivalent number of moles of plasmids AGP73, AGP81, 2380, AGP82, AGP83, AGP212, and AGP213, were co-transfected with 1 μg of 2145 into 1 × 10^7 ^293/EBNA1 cells on a 10 cm dish. Cells were split eight hours post-transfection so that they would not be confluent at 48 hours post-transfection, at which time cells were harvested, FACS profiled to measure GFP expression, and re-plated in duplicate at 2 × 10^5^, 2 × 10^4^, and 2 × 10^3 ^GFP-positive, live cells per culture dish. Cells were placed under selection with 0.5 μg/ml puromycin four days post-transfection. After two weeks of selection, the resulting puromycin-resistant colonies were fixed with formamide and subsequently stained with methylene blue. Colonies that were at least 2 mm in size were scored as positive. Colonies were counted using a colony counting macro written for NIH Image as described previously [17,18].

### Southern hybridization analysis to assess plasmid replication

Ten μg of AGP74 or an equivalent number of moles of plasmids AGP73, 2380, AGP212, and AGP213 were co-transfected with 1 μg of 2145 into 1 × 10^7 ^293/EBNA1 cells on a 10 cm dish. Cells were placed under puromycin selection 48 hours post-transfection. After three weeks of selection, episomal DNAs were extracted from cells in puromycin resistant colonies that were pooled. Episomal DNAs were extracted from 2 × 10^7 ^– 10^8 ^puromycin-resistant cells as described previously [11,30]. Extracted DNAs were digested with 200 units of *Dpn*I, 20 units of *Bam*HI, and 20 units of *Xba*I in a final volume of 100 μl overnight at 37°C. Restriction endonucleases were purchased from New England Biolabs (Beverly, MA), and used as per the manufacturer's instructions. Digestions were extracted with phenol:chloroform (1:1), precipitated and electrophoresed on a 0.8% agarose gel. DNAs were transferred from the gel to Hybond membrane (Amersham, Buckinghamshire, UK) using an Appligene vacuum transfer apparatus (Boekel Scientific, Feasterville, PA). Radioactive probes were prepared by the incorporation of α-^32^P-dCTP (6000 Ci/mmol) (Amersham) during Klenow synthesis using random primers and *Pst*I-digested AGP83 as template. Probe specific activities ranged from 1 × 10^9 ^cpm/μg to 3 × 10^9 ^cpm/μg. Southern hybridization was performed as described by Hubert and Laimins [31,32]. Southern blots were visualized and quantified by phosphorimage analysis using a Molecular Dynamics Storm phosphorimager (Molecular Dynamics, Sunnyvale, CA).

### Transfection of linear plasmid DNAs to assess replication fork migration in vivo

Ten μg of *Pvu*II-linearized AGP73 or AGP74 was transfected into 293/EBNA1 cells as described above along with 1 μg of 1606. Hirt extracts were prepared from 2 × 10^7 ^transfected cells 14 – 16 hours post-transfection and digested exhaustively with *Dpn*I (200 units). The digested extracts were then digested with *Hin*dIII (10 units) &*Acc*65I (10 units) to release a 1063 bp fragment between the SV40 origin and FR, and with *Bsr*GI (10 units) &*Spe*I (20 units) to release a 637 bp fragment that lies immediately after FR. The digested products were separated on a 1.5% agarose gel electrophoresed in 0.5× TBE, and transferred to Hybond membrane and probed as described above. Probe was synthesized using random primers and the *Hin*dIII-*Acc*65I fragment, as well as the *Bsr*GI-*Spe*I fragment as template. In control experiments, probes were hybridized against purified fragments to confirm that the *Bsr*GI-*Spe*I fragment bound approximately two-thirds as much probe as the *Hin*dIII-*Acc*65I fragment.

### Transcription reporter assays

100 ng of pRSVL [33], or an equivalent number of moles of AGP39, AGP40, or AGP41 was co-transfected with 1 μg of 2145 and 10 μg of pcDNA3 or 10 μg of 1160 into 293 cells. Cells were split eight hours post-transfection so that they would not have reached confluence when harvested 72 hours post-transfection. A fraction of the harvested cells were then counted twice using a Coulter counter, and FACS profiled to normalize for the fraction of live transfected cells. The remainder of the cells were pelleted, and lysed in reporter lysis buffer (provided along with a luciferase assay kit from Promega, Madison, WI) at a concentration of 1 × 10^5 ^cells/μl. Lysates were spun for 5 minutes at 1000 g to remove nuclei, and then frozen at -80°C until assay. Luminescence assays were performed as per manufacturer's instructions, using a Zylux FB 15 luminometer.

### RT-PCR analysis to measure migration of transcription forks through FR

Total RNA was extracted from transfected 293 cells using the SV Total RNA Isolation System from Promega (Madison, WI). PolyA+ RNA was extracted from transfected 293 cells using the PolyATract mRNA Isolation System from Promega (Madison, WI). Either 5 μg of total RNA or 1 μg of polyA RNA was used in RT-PCR reactions using the following primers to detect firefly luciferase:

AGO83: 5' GGAATACTTCGAAATGTCCG

AGO84: 5' TCATTAAAACCGGGAGGTAG

Control RT-PCR reactions amplifying the glyceraldehyde phosphate dehydrogenase (GAPDH) transcript were performed using the following two primers:

AGO81: 5' CTCAGACACCATGGGGAAGGTGA

AGO82: 5' ACTTGATTTTGGAGGGATCTCG

RT-PCR reactions were performed using the AccessQuick one-tube RT-PCR System purchased from Promega (Madison, WI).

## Results

### The number of viable colonies decreases with an increasing number of EBNA1 binding sites in FR

During our studies to determine the optimal number of binding sites in FR, as well as the spacing between adjacent sites, we determined that plasmids with ten high-affinity EBNA1 binding sites in a synthetic FR formed puromycin resistant colonies in 293/EBNA1 that were 2 mm in size and larger more efficiently than colonies with 20 binding sites in FR [24]. The EBNA1-binding sites in the synthetic FRs are identical, and were chosen using the sequence of the EBNA1-binding site found most frequently in the natural FR (seven times out of 20) (*ibid*). There is a small amount of sequence variation between binding sites in the natural FR. The most frequent site is repeated seven times, and an additional 11 sites are single nucleotide variations of this site [6,34]. To eliminate the possibility that an FR with 20 identical EBNA1-binding sites behaves differently than the natural FR, we compared the colony formation efficiency of plasmids containing a synthetic FR with 20 binding sites versus the natural FR, and found their efficiencies to be indistinguishable (Table 1). Therefore our observation that plasmids with ten identical EBNA1-binding sites in FR form colonies more efficiently than plasmids 20 identical EBNA1-binding sites in FR recapitulates the observations made with natural FR, or deletion derivatives thereof [25]. These authors have determined that a plasmid containing a deletion mutation of the natural FR with only nine EBNA1-binding sites replicates more efficiently than a plasmid with the intact natural FR containing 20 binding sites (*ibid*). Next, it was determined whether additional increases in the number of EBNA1-binding sites would continue to decrease the efficiency of replication and therefore decrease the number of colonies formed. For this, the colony formation efficiency of reporter plasmids with FRs containing 40 and 80 binding EBNA1-binding sites was measured in 293/EBNA1 cells. The results of this assay are summarized in Table 1. The summarized results indicate several observations: 1) In concordance with our previous results, plasmids with ten binding sites in FR form colonies approximately four times more efficiently than colonies with 20 binding sites in FR. This difference is statistically significant with a *p*-value of 0.02 by the Wilcoxon rank-sum test; 2) The FR with 20 identical EBNA1-binding sites cannot be distinguished statistically from the natural FR in colony formation assays and replication assays (see below); 3) Most surprisingly, the efficiency of colony formation decreases sharply for replication reporters containing FRs with 40 or 80 EBNA1-binding sites, such that a plasmid with 40 binding sites in FR formed puromycin-resistant colonies approximately one log less efficiently than a plasmid with 20 binding sites in FR, and a plasmid with 80 binding sites in FR formed colonies two logs less efficiently than a plasmid with 20 binding sites in FR. Both these decreases were found to be highly significant (*p *< 0.01 by the Wilcoxon rank-sum test). Indeed a plasmid with 80 EBNA1-binding sites formed 2 mm and larger colonies with the same efficiency as replication reporter that only contained the DS element (Table 1).

**Table 1:** A greater than wild-type number of EBNA1 binding sites in the family of repeats causes a decrease in the number of puromycin resistant colonies obtained in colony formation assays.

**Replication reporter transfected**	**Number of EBNA1 binding sites in FR^a^**	**Colonies per 10^5 ^live, transfected cells plated^b^**
AGP83	0	2 ± 1.8
AGP73	10	4390 ± 311.1
AGP74	20	1296 ± 106.1
2380	20^c^	1230 ± 28.3
AGP81	40	78 ± 14.2
AGP82	80	3 ± 1.7

^a ^plasmids contain DS, EBV sequences between FR and DS, and a synthetic FR containing the indicated number of EBNA1 binding sites.

^b ^puromycin resistant colonies present 18 days post-transfection that are 2 mm and larger in size.

^c ^plasmid 2380 contains wild-type FR from the B95-8 strain of EBV.

**Figure 1:**
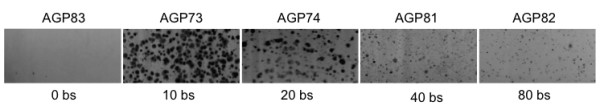
**Plasmids with an FR containing more than 20 EBNA1 binding sites form minute colonies under selection**. The indicated plasmids were transfected into 293/EBNA1 cells, which were subjected to puromycin selection for 18 days in colony formation assays as described in the Materials and Methods section. Representative images of methylene blue stained colonies are shown. The identity of the transfected plasmid is indicated above each image, and the number of EBNA1 binding sites present in the FR of each plasmid is indicated below each image. As a negative control, an assay was also performed with AGP83, a DS-only plasmid that replicates transiently, but is not partitioned, and forms colonies with very low efficiency. The colonies formed from cells transfected with AGP81 and AGP82 did not increase in size even after several weeks of growth in selective media. The number of colonies formed in such assays that were 2 mm in size and larger is indicated in Table 1.

The colony formation assay we employ only counts colonies that are 2 mm or larger in size by 18 days post-transfection. We observed that 293/EBNA1 cells transfected with replication reporter plasmids containing 40 or 80 binding sites in FR formed a large number of colonies that were substantially smaller than 2 mm in size, and never increased in size despite two additional weeks of growth in selective media (Figure 1, and data not shown). Figure 1 contains examples of colony formation assays performed with plasmids that contain only DS, or DS with increasing numbers of EBNA1-binding sites in FR. As seen in the figure, while a plasmid containing DS alone forms very few colonies, plasmids with 40 or 80 EBNA1-binding sites in FR form a large number of colonies that are much smaller than 2 mm in size. In contrast, the majority of colonies formed by cells transfected with plasmids containing ten or 20 EBNA1-binding sites in FR are larger than 2 mm in size.

The large number of tiny colonies formed upon transfection of plasmids containing 40 or 80 binding sites in FR is consistent with the behavior of plasmids that confers puromycin resistance to transfected cells but are not distributed to daughter cells at mitoses, thus preventing the formation of a large puromycin-resistant colonies. This could happen either due to a defect in plasmid partitioning or due to a failure in plasmid replication. We favor a defect in plasmid replication, because the colony formation phenotype of these two plasmids is strikingly different from that of a plasmid containing only DS (Figure 1). DS-only plasmids are replicated transiently but not partitioned, and thus give rise to a few puromycin-resistant colonies that contain integrated copies of the plasmid [24]. For the reporter plasmids containing 40 and 80 EBNA1-binding sites in FR, the presence of a large number of colonies that do not expand in size suggests that the initially transfected plasmids are partitioned, but are poorly replicated, if at all. Therefore, the cells that nucleate a colony cannot give rise to drug-resistant daughters upon cell proliferation, as the latter lack plasmids to confer drug resistance. In this study we have examined why increasing the number of EBNA1-binding sites in FR decreases the efficiency of plasmid replication.

**Figure 2:**
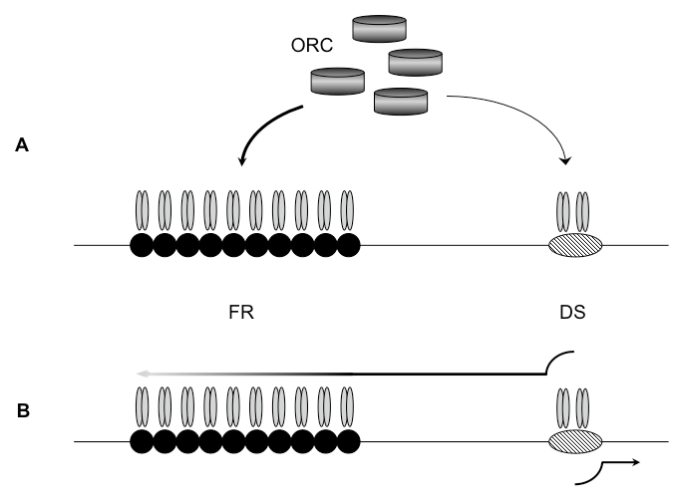
**Two models to explain decreases in copy number when *oriP *plasmids contain an FR with 20 or more EBNA1 binding sites**. DS is represented as a striped oval, and EBNA1-binding sites in FR are represented as black filled circles. For simplicity, only ten binding sites are shown. EBNA1 dimers bound to DS or FR are represented as gray ovals. (A) Replication factor titration model. EBNA1 bound to FR is proposed to non-functionally titrate cellular replication factors, such as ORC proteins, away from EBNA1 bound to DS, thus decreasing replication initiation events at DS. The titration efficiency is proportional to the amount of EBNA1 at FR, which in turn is dependent on the number of EBNA1 binding sites in FR. (B) The replication fork barrier model in which EBNA1 bound to FR is proposed to act post-initiation to impede the progression of replication forks initiated at DS. A decreased efficiency of progression is indicated by the gradation in line color from black to light gray. The strength of this barrier is proportional to the amount of EBNA1 present at FR, which is also dependent on the number of EBNA1 binding sites in FR.

There are two possible reasons for this defect, illustrated by the models in Figure 2. In Figure 2A we have schematically depicted the "replication factor titration" model proposed earlier [25]. In this model, EBNA1 bound to FR is proposed to non-functionally titrate cellular replication factors, such as the ORC proteins, away from EBNA1 bound to DS. This non-functional recruitment of proteins such as ORC decreases the replication potential of plasmids, by reducing the frequency of replication initiation as DS, as the number of EBNA1-binding sites in FR is increased. An alternative model is suggested by the results of Gahn and Schildkraut [22], who have demonstrated that FR forms a barrier that attenuates the migration of replication forks initiated at DS. If the efficiency of attenuation is dependent upon the number of EBNA1-binding sites in FR, then increases in binding site number are predicted to decrease replication efficiency. Thus in this model, termed the "replication fork barrier" model, and depicted in Figure 2B, EBNA1 bound to FR suppresses replication from DS by attenuating the migration of the replication fork after initiation of DNA synthesis. If this latter model underlies the relative inefficiency in the replication of plasmid with 20 binding sites compared to a plasmid with ten binding sites, then it is predicted that presenting the 20 binding sites as two widely-separated sets of ten binding sites on a plasmid should revert the observed decrease. On the other hand, the replication factor titration model predicts that a plasmid with two FRs, each with ten binding sites, should replicate with the same efficiency as a plasmid with a single FR containing 20 EBNA1-binding sites.

### Replication of plasmids with split FRs containing ten binding sites each

**Figure 3:**
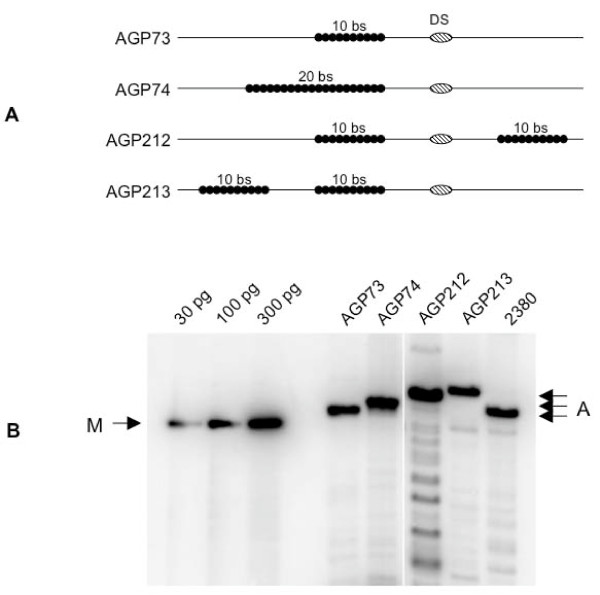
**Split FRs distinguish between the replication factor titration and replication fork barrier models**. (A) Schematic representation of the *oriP *region from plasmids designed to distinguish between the replication factor titration and the replication fork barrier models. The identity of the plasmid is indicated to the left of each schematic. DS is represented as a striped oval, and the EBNA1-binding sites in FR as filled black circles. The number of EBNA1 binding sites within each FR is indicated above each FR. FRs are separated from each other (plasmid AGP213) or from DS (plasmid AGP212) by the EBV sequences normally present between FR and DS. (B) Stable replication of *oriP *replication reporters under selection in 293/EBNA1 cells. 293/EBNA1 cells were transfected with the indicated plasmid, placed under puromycin selection for 18 days, at which time replicated *Dpn*I-resistant episomal DNAs were recovered and quantified as described in the Methods. "M" indicates the migration position of standards used for quantitation, and the amounts of standards loaded are indicated above each lane. The identity of the transfected plasmid is indicated above each lane. "A" indicates the migration position of *Dpn*I-resistant, linearized plasmid DNAs.

To test the models presented in Figure 2, two additional replication reporter plasmids illustrated in Figure 3A were constructed. In the first, AGP212, two FRs with ten binding sites each were placed on either side of DS, and separated from DS by the EBV sequences normally present between FR and DS. In the second, AGP 213, two FRs with ten binding sites each were placed in tandem, but separated from each other by the EBV sequences normally present between FR and DS. AGP212 and AGP213 were transfected into 293/EBNA1 cells and their ability to form puromycin resistant colonies was evaluated (Table 2). As indicated in the table, both plasmids containing 20 EBNA1-binding sites split into two sets of ten binding sites form puromycin resistant colonies far more efficiently than a plasmid containing 20 contiguous EBNA1 binding sites in FR, or a plasmid that contains wild-type FR (*p*-value < 0.05 by the Wilcoxon rank-sum test). Not only do AGP212 and AGP213 form colonies more efficiently than AGP74, they also give rise to puromycin-resistant colonies more efficiently than AGP73 that contains a single block of ten EBNA1-binding sites. This result favors the "replication fork barrier" model over the "replication factor titration" model.

**Table 2:** Splitting twenty contiguous EBNA1 binding sites into two sets of ten binding sites increases the efficiency of replication as estimated by colony formation.

**Replication reporter transfected**	**Arrangement of EBNA1 binding sites^a^**	**Colonies per 10^5 ^live, transfected cells plated^b^**
AGP73	FR(10), DS	4390 ± 311.1^c^
AGP74	FR(20), DS	1296 ± 106.1^c^
AGP212	FR(10), DS, FR(10)	10048 ± 371.7
AGP213	FR(10), FR(10), DS)	6132 ± 180.2

^a ^The arrangement of EBNA1 binding sites in FR is schematically depicted in Figure 3.

^b ^puromycin resistant colonies present 18 days post-transfection that are 2 mm and larger in size.

^c ^values are taken from Table 1, and shown here for convenience,

To verify that AGP212 and AGP213 are replicated episomally, episomal DNA from 293/EBNA1 cells transfected independently with AGP73, AGP74, 2380, AGP212 and AGP213, was extracted 18 days post-transfection, digested exhaustively with *Dpn*I, linearized with *Xba*I, and examined by Southern blot. These results are shown in Figure 3B, and tabulated in Table 3. These results indicate that all the plasmids are replicated episomally and maintained at copy numbers varying between ~25 and ~80 molecules per transfected cell under selection.

**Table 3:** Copy number of replicated, *Dpn*I-resistant, plasmids detected 18 days after transfection into 293/EBNA1 cells.

**Replication reporter transfected**	**Arrangement of EBNA1 binding sites**	**Plasmid copy number**
AGP73	FR(10), DS	38 ± 11
AGP74	FR(20), DS	47 ± 8
AGP212	FR(10), DS, FR(10)	60 ± 11
AGP213	FR(10), FR(10), DS	51 ± 11
2380	wild-type *oriP*	44 ± 9

^a ^Numbers represent the average number of *Dpn*I-resistant episomal plasmid molecules per transfected cell detected in three experiments along with the standard deviation.

### EBNA1 bound to 20 contiguous binding sites in FR impedes the migration of replication forks within cells

**Figure 4:**
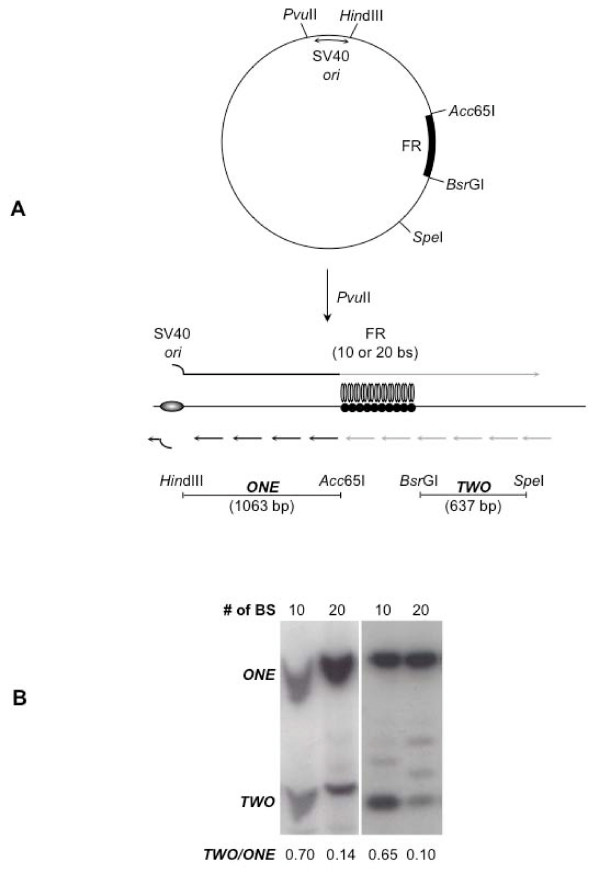
**EBNA1 bound to FR blocks the progression of replication forks in transfected cells**. A) Plasmids containing the SV40 replication origin, and FR regions with ten or 20 EBNA1-binding sites were linearized, and co-transfected into 293/EBNA1 cells with large T-antigen expression plasmid. A schematic representation of bidirectional replication fork movement from the SV40 origin is indicated above and below the linear transfected DNA, with the position of FR and the SV40 origin indicated. The leading strands from the SV40 origin are indicated as long arrows, and Okazaki fragments as the short arrows. Dark lines indicate unimpeded fork progression, while light gray lines indicated segments where diminished DNA synthesis is predicted. The positions and identities of restriction enzyme recognition sites to liberate fragments "ONE" and "TWO" from replicated DNA are shown. (B) Hirt extraction was sued to recover DNAs from transfected 293/EBNA1 cells that were subsequently digested with *Dpn*I and the specified restriction endonucleases to release fragments ONE and TWO, which were separated by electrophoresis, and quantified by Southern blot. Two independent experiments are shown with the migration of fragments ONE and TWO, and the number of EBNA1-binding sites in FR indicated. The TWO:ONE ratio is also shown.

The results described above are interpreted to indicate that EBNA1 bound to 20 contiguous binding sites limits replication from *oriP *in a manner consistent with it impeding the migration of replication forks. To determine whether the efficiency with which EBNA1 bound to FR impedes replication fork migration is dependent upon the number of binding sites in FR, the experiment schematically depicted in Figure 4A was performed. 293/EBNA1 cells were co-transfected with linear DNAs containing the SV40 origin, and FRs with either ten or 20 EBNA1-binding sites, along with a large T-antigen expression plasmid. Fourteen to 16 hours post-transfection, low molecular DNAs were recovered by the method of Hirt and digested exhaustively with *Dpn*I to remove any unreplicated linear DNAs present from the transfection. The *Dpn*I-treated DNA was then digested with *Hin*dIII and *Acc65*I to release a 1 kb fragment (labeled fragment ONE) that lies immediately between the SV40 origin and FR, and with *Bsr*GI and *Spe*I to release a 0.6 kb fragment (labeled fragment TWO) that lies immediately after FR. The digested DNAs were electrophoresed on a 1.5% agarose gel, transferred to nylon and probed for each of the fragments. If EBNA1 bound to FR does not function as block to the migration of replication forks from the SV40 origin *in vivo*, we expect that equivalent amounts of fragment ONE and TWO will be synthesized. In contrast, if EBNA1 bound to FR efficiently blocks replication forks from the SV40 origin *in vivo*, we expect a smaller amount of *Dpn*I-resistant, replicated fragment TWO relative to fragment ONE. It is pertinent to note that because fragment TWO is smaller than fragment ONE, a TWO/ONE ratio of approximately 0.6 is indicative of equivalent amounts of both fragments. The results of two independent experiments are shown in Figure 4B. As can be seen from the Figure, when FR in the transfected plasmid contained ten EBNA1 binding sites, the TWO/ONE ratio averaged 0.67, indicating that pieces of DNA on either side of FR were synthesized equivalently. In contrast, when FR contained 20 binding sites, the TWO/ONE ratio averaged 0.12, indicating that the fragment before FR was synthesized five-times as much as the fragment after FR. This experiment provides strong *in vivo *molecular evidence that EBNA1 bound to 20 contiguous binding sites attenuates the migration of replication forks. Further, the strength of attenuation is dependent upon the number of binding sites for EBNA1, and is non-existent when only ten contiguous binding sites are on the template.

Thus, we conclude that plasmids containing the wild-type number of binding sites in FR are replicated less well than plasmids with fewer binding sites in FR (Table 1, Figure 1, Figure 4). The apparent conundrum posed by this data is to explain why the EBV genome has evolved to contain a plasmid-partitioning element that reduces the efficiency with which the genome is replicated. One possible reason for this is that it provides a mechanism for EBV to limit the replication of its latent replicon and maintain copy number control in latently infected cells. An increase in genome copy number may result in the unfettered expression of viral genes, and thereby compromise the ability of latently infected cells to evade immune surveillance. We believe it likely that there are additional reasons that EBNA1 bound to FR attenuates fork migration. It has been demonstrated that plasmids with active transcription units suppress the use of replication origins on the same plasmid [35,36]. This could possibly arise from the collision of transcription and replication forks on the same plasmid, resulting in the faster transcription forks stalling the slower migrating replication forks [37-40], possibly generating of double-strand breaks (DSBs) [41]. In its natural context, *oriP *is immediately adjacent to the EBER genes that are heavily transcribed during latency, which is also when *oriP *is active as a replication origin. The EBERs are transcribed toward DS, the replication origin within *oriP*, and separated from DS by FR. Therefore, we wished to test whether EBNA1 bound to FR could terminate the migration of transcription forks, and thereby protect replication forks initiated at DS.

### EBNA1 bound to FR impedes the progression of transcription forks

**Figure 5:**
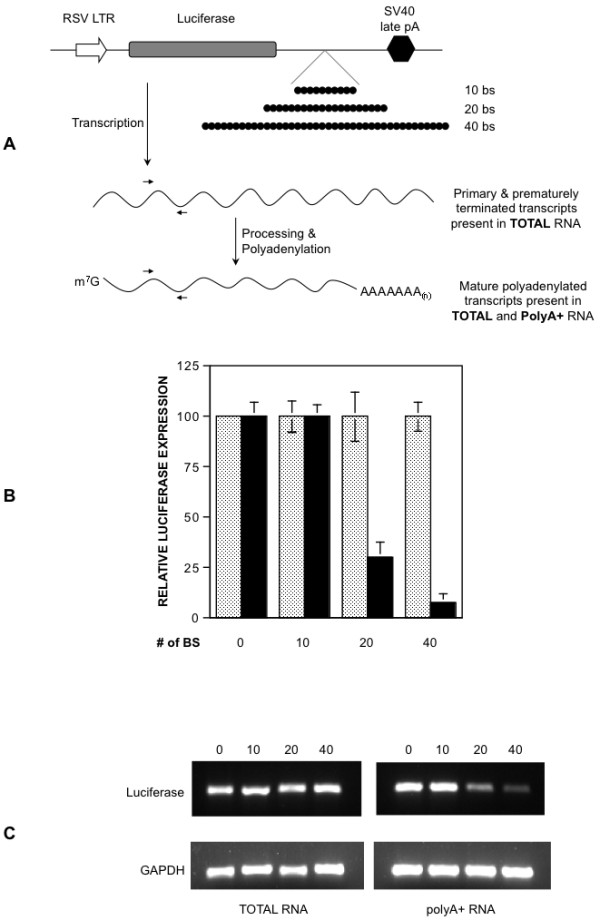
**EBNA1 bound to FR impedes transcription fork progression**. (A) Representation of transcription reporter plasmids used here. In pRSVL, the RSV LTR drives transcription of the luciferase gene, and the SV40 late polyadenylation signal is used for polyadenylation. Derivatives of pRSVL with ten, 20 or 40 EBNA1-binding sites (filled black circles) between the luciferase gene and the polyadenylation signal were constructed. Primary transcripts, and prematurely terminated transcripts present in total RNA preparations are indicated, as are mature luciferase mRNAs present in total and polyA+ RNA preparations. Primers used for RT-PCR are indicated as arrows. (B) Luciferase expression from the reporter plasmids described above. Plasmids were co-transfected with either pcDNA3 (stippled bars), or a EBNA1 DNA binding domain expression plasmid (black bars) into 293 cells. The number of EBNA1 binding sites in the reporter plasmid is indicated below each pair of bars. Luciferase activity is reported relative to the activity observed when pRSVL was co-transfected with pcDNA3. (C) RT-PCRs to detect luciferase and GAPDH transcripts in total or polyadenylated RNAs recovered from the transfected cells described in B. PCR products were visualized with ethidium bromide and the identity of the transfected plasmid is indicated above each lane.

The transcription reporter plasmid pRSVL [33] was modified to introduce ten, 20, or 40 contiguous EBNA1 binding sites between the end of the luciferase open reading frame and the SV40 polyadenylation sequence in that plasmid. The structure of these reporter plasmids is shown in Figure 5A. These reporter plasmids were then co-transfected into 293 cells with a control expression plasmid (pcDNA3), or plasmid 1160 that expresses the DNA binding domain of EBNA1 (DBD). Cells were harvested two days post-transfection, FACS profiled to normalize for live transfected cells, following which luciferase levels were measured. This analysis is shown in Figure 5B. In the absence of EBNA1 binding sites on the reporter plasmid, the co-transfected DBD expression plasmid had no effect on luciferase expression. Similarly, pcDNA3 had no effect on luciferase expression from reporter plasmids that contained ten, 20 or 40 EBNA1 binding sites. However when the luciferase reporter plasmids had 20 or 40 EBNA1 binding sites, and were co-transfected with the DBD expression plasmid, there was a sharp decrease in the expression of luciferase dependent upon the number of number binding sites placed 5' to the polyadenylation signal.

It was speculated that this decrease in luciferase expression resulted from prematurely terminated luciferase transcripts formed as a consequence of DBD bound to EBNA1 binding sites functioning as a transcription fork-block. To test this hypothesis, the distribution of luciferase RNA in total and polyA+ RNA pools was examined by reverse-transcriptase PCR (RT-PCR), with the following rationale. Prematurely terminated transcripts should be transiently detected in the total RNA pool but not the polyA+ pool, while the mature luciferase mRNA should be present in both pools of RNA. The rationale is depicted schematically in Figure 5A, and the experimental outcome is shown in Figure 5C. As seen in the figure, the DBD did not effect amplification of the target sequence by RT-PCR from both the total RNA and mRNA pools when cells were transfected with pRSVL, or a derivative of pRSVL containing ten EBNA1-binding sites before the polyadenylation signal. In contrast, for derivatives of pRSVL containing 20 or 40 EBNA1-binding sites before the polyadenylation signal, there was a clear decrease in amplification of the luciferase target sequence by RT-PCR from polyA+ RNA pool, mirroring the decrease in luciferase expression observed in Figure 5B. However, the target was amplified from the total RNA pool recovered from cells transfected with these plasmids (*ibid*). We interpret this analysis to indicate that the decrease in luciferase signal observed in Figure 5B results from EBNA1 bound to FR acting to terminate the migration of transcription forks, and that this termination can be observed when FR contains the wild-type number of 20 binding sites, but not ten binding sites.

## Discussion

In this study we have demonstrated that the wild-type number of EBNA1-binding sites in EBV's FR region is sub-optimal for the efficient replication of *oriP*-plasmids. A plasmid with ten binding sites in FR formed colonies more efficiently than a plasmid with the wild-type number of 20 binding sites. Increasing the number of binding sites in FR beyond 20 further decreased the efficiency of replication (Table 1). These results corroborate those of Leight and Sugden who have demonstrated that an *oriP*-plasmid with a deletion that removes approximately one-half of FR is replicated more efficiently than a plasmid with wild-type FR [25].

We have tested two models to explain why plasmids with fewer binding sites in FR are replicated more efficiently than plasmids with the wild-type number of binding sites. Our results support a model wherein EBNA1 bound to FR impedes the progression of replication forks that originate from DS. It was determined that this effect correlates with the number of contiguous EBNA1 binding sites in FR. Attenuation of fork migration is not readily detected with ten contiguous sites, but is easily observed with 20 contiguous binding sites. As has been observed previously *in vitro *[23], we found that EBNA1 bound to FR also impedes replication forks from the SV40 origin within transfected cells. The SV40 origin was used for this analysis because it fires multiple times in a single cell-cycle, permitting facile evaluation of the reduction in fork migration. The major difference between the SV40 replication fork and replication forks that initiate from DS lies in the nature of the leading strand helicase. The hexameric large T-antigen helicase in the SV40 replication fork has approximately the same mass as the hexameric MCM helicase present at replication forks that initiate from DS [7]. Both forks progress at similar rates, with elongation being estimated at approximately 100 bp/min for the SV40 replication fork [42,43], and at between 10 – 50 bp/min for EBV replication [44]. Given the similar biophysical characteristics of both forks, we believe that EBNA1 bound to FR will impede the progression of replication forks that fire from DS in a manner dependent on the number of binding sites.

Our data indicates that split-FR plasmids containing two FRs with ten binding sites each are replicated more efficiently than plasmids containing a single FR with twenty contiguous EBNA1 binding sites (Table 2). EBNA1 bound to FR tethers *oriP*-plasmids to chromosomes to facilitate their maintenance and partitioning in proliferating cells [17-19]. The efficiency of this process is dependent upon the number of binding sites in FR, such that an EBNA1 mutant which is partially defective in chromosomal association can be rescued by increasing the number of binding sites in FR [18]. However, with 20 contiguous sites, this increase in partitioning efficiency is offset by a decrease in replication efficiency. Splitting the 20-binding site FR into separated FRs with ten binding sites each, no longer impedes replication, but retains the advantage of having 20 binding sites for efficient *oriP*-plasmid partitioning. The data obtained with AGP212 and AGP213 (Table 2) also indicates that the replication factor titration model proposed previously is unlikely. Both these plasmids contain 20 EBNA1 binding sites and replicate more efficiently than a plasmid that contains ten binding sites. Were the titration model to be correct, replication of these plasmids would be less efficient than replication of a plasmid with ten EBNA1 binding sites in FR.

The ability of EBNA1 to impede replication fork migration likely impacts replication of EBV genomes. Besides DS, there are other replication origins on the EBV genome also used during latency [45], such as an origin that lies in the *Bam*HI-A fragment [46,47]. It is known that collision of replication forks can lead to fork collapse, and the consequential generation of double-stranded breaks (DSBs) [48]. Such events can lead to irregular recombination events, and a large number of DSBs causes apoptosis [49-51]. We propose that EBNA1 bound to FR acts as a buffer to prevent two replication forks from running into each other and thereby protects cells latently infected by EBV from undergoing apoptosis as a consequence of DSB generation.

There is a striking parallel between the function of EBNA1 at FR and TTFI at *Sal *repeats that terminate ribosomal DNA replication. Both proteins impede the progression of replication forks dependent on the number of binding sites for the protein on the template DNA [52]. Additionally, just as TTFI bound to the *Sal *repeats blocks the progression of transcription forks and terminates them [53], we have found that EBNA1 bound to FR blocks the progression of transcription forks in a manner dependent upon the number of binding sites (Figure 5). In its natural chromosomal context the ribosomal DNA replication fork block is required for the proper termination of rRNA transcripts. Within the EBV genome, the EBER RNA genes are immediately 5' of FR and transcribed toward it [34]. The EBERs are pol III transcripts [54,55], and it is now known that some cellular pol III transcripts are terminated by pol II transactivators acting as transcription fork blocks [56]. On this basis, we speculate that EBNA1 bound to FR participates in the proper termination of EBER RNAs. It is also possible that FR prevents transcription forks emanating from the EBER genes colliding with replication forks emanating from DS. Similar to collisions between replication forks, such collisions also cause replication-fork collapse, with the consequent pro-apoptotic generation of DSBs.

In conclusion, there are several reasons for EBV to have an FR that is sub-optimal for plasmid replication. It is clear that EBNA1 bound to FR activates transcription from multiple viral promoters [57-59], a property of EBNA1 necessary for naïve B-cells to be immortalized by EBV [60]. We and others have demonstrated that ability of EBNA1 to activate transcription is proportional to the number of binding sites in FR [24,61]; EBNA1 bound to 20 binding sites activates transcription approximately two to three times as well as EBNA1 bound to ten binding sites [24]. Thus, while the number of EBNA1-binding sites in FR is sub-optimal for replication of *oriP*-plasmids, this number of binding sites is likely necessary for EBNA1 to transactivate effectively. It is also intriguing that when bound to 20 binding sites, EBNA1 functions effectively as a transcription and replication fork-block, leading us to conjecture that the latter activity protects latently infected cells by preventing DNA damage resulting from collisions between a replication fork originating at DS, and transcription or replication fork-blocks emanating from elsewhere in the EBV genome.

## Conclusion

We conclude from this data that upon binding FR, EBNA1 limits the replication of *oriP*-plasmids by impeding the progression of replication forks through FR. The impedance is dependent on the number of EBNA1-binding sites within FR, and is observed with the wild-type number of binding sites. Splitting the wild-type number of binding sites in FR into two sets of ten binding sites creates *oriP*-plasmids that maintained up to ten-fold more efficiently than wild-type *oriP*-plasmids. EBNA1 bound to FR also impedes the progression of transcription forks through FR. This data permits us to propose that in addition to limiting the replication of EBV genomes during latency, EBNA1 bound to FR may prevent the formation of double-stranded breaks as a consequence of fork collision.

## Competing interests

The authors declare that they have no competing interests.

## Authors' contributions

AA was responsible for experimental design, conducting experiments, and writing the manuscript. SA was responsible for experimental design, and conducting experiments. AW was responsible for conducting experiments. GS was responsible for conducting experiments. RBL was responsible for experimental design, and writing the manuscript.
